# Mechanical characterization of human umbilical and chorionic plate arteries affected by fetal growth restriction

**DOI:** 10.1093/pnasnexus/pgag158

**Published:** 2026-05-07

**Authors:** Germán A Arenas, Álvaro Navarrete, José Miguel Gonzalez, Andrés Utrera, Claudio García-Herrera, Bernardo J Krause

**Affiliations:** Facultad de Ciencias Biológicas, Pontificia Universidad Católica de Chile, Santiago 8331150, Chile; Departamento de Ingeniería Mecánica, Universidad de Santiago de Chile, Santiago de Chile 9170016, Chile; Facultad de Medicina, Pontificia Universidad Católica de Chile, Santiago 8331150, Chile; Departamento de Ingeniería Mecánica, Universidad de Santiago de Chile, Santiago de Chile 9170016, Chile; Facultad de Inegniería y Ciencias, Universidad Adolfo Ibañez, Santiago 7910000, Chile; Instituto de Ciencias de la Salud, Universidad de O’Higgins, Rancagua 2820000, Chile

**Keywords:** fetoplacental arteries, biomechanical characterization, ring-tensile test, ring-opening test, finite element method

## Abstract

Fetal growth restriction (FGR) is a pregnancy complication associated with increased perinatal morbidity and mortality in the short term, along with an elevated risk of developing cardiometabolic diseases in the long term. FGR is also associated with vascular dysfunction in the fetoplacental unit. In this work, we develop a clinical study of the fetoplacental circulation (encompassing umbilical and chorionic arteries) under the FGR condition, utilizing a combination of numerical and experimental approaches to quantify biomechanical and morphological characteristics. Placental samples from normal (*n* = 5) and FGR pregnancies (*n* = 5) underwent biomechanical testing (ring-opening and ring-tensile tests) under physiological conditions. Biomechanical behavior, including material properties and residual stress, was characterized via numerical analysis using a hyperelastic model and a simulation of the ring-closure process. Morphological analysis, including wall thickness and layer area measurements, was performed to relate structural features to biomechanical behavior. The umbilical and chorionic arteries exhibit distinct responses to FGR: the umbilical artery undergoes both morphological remodeling and changes in biomechanical properties, whereas the chorionic artery primarily shows biomechanical alterations. Overall, this study provides novel biomechanical evidence of the impact of FGR on placental vasculature, highlighting the complex interplay between morphology and mechanics in fetoplacental blood vessels.

Significance Statement Fetal growth restriction (FGR) is associated with short- and long-term health risks, yet its biomechanical impact on the fetoplacental circulation remains poorly understood. This study investigates these effects using a combined experimental and numerical approach, supported by morphological analysis. Umbilical and chorionic arteries were extracted from normal and FGR placentas. Ring-tensile and ring-opening tests assessed the stress–stretch relationship and residual stress levels. Results show biomechanical alterations in both arteries under FGR, with additional morphological changes in the umbilical arteries. These findings align with previous ultrastructural evidence and suggest that FGR leads to vascular remodeling. This work offers new insights into placental biomechanics, contributing to a better understanding of FGR-related vascular dysfunction.

## Introduction

Fetal growth restriction (FGR) is a pathological pregnancy complication that affects ∼5–10% of pregnancies (1, 2) and is associated with increased perinatal morbidity and mortality (3) and a higher lifetime risk of cardiometabolic disease (4, 5). FGR is also associated with both maternal disease and fetoplacental vascular dysfunction (6, 7). Clinically, FGR is defined as an estimated fetal weight below the 10th percentile for gestational age, with abnormal umbilical artery Doppler velocimetry serving as an important indicator of impaired fetoplacental circulation (8). Because the fetoplacental circulation supplies oxygen and nutrients to the fetus, functional and structural disturbances of this vascular circuit are thought to underline the vascular dysfunction observed in FGR. Moreover, vascular adaptations that occur during fetal development influence later cardiovascular disease risk (9), supporting the crucial importance of studying its early structural effects.

In this context, biomechanics enables the study of complex interactions between physical constraints and physiological processes within the circulatory system (10). Solid biomechanics, for example, focuses on the vascular wall and its structural organization (11) and is increasingly applied to the assessment of vascular impairments associated with aging (12), as well as the development and progression of cardiovascular diseases (13–15). From a biomechanical perspective, several studies using animal models have examined the effects of FGR on alterations in the mechanical properties of blood vessels, primarily within the systemic circulation (11, 16, 17). The passive elastic response, mainly determined by the extracellular matrix (18), has been evaluated through different experimental approaches, such as uniaxial tensile (19), ring tensile (20), and pressure-inflation (21, 22) tests. Additionally, the residual strain on vessels, influenced by the prestretched state of the elastin (23), can be assessed through the ring-opening test (20). The passive viscoelastic response, characterized by the action of collagen fibers, proteoglycans, and their respective interaction (24), has been examined through stress relaxation tests (19). However, accurate interpretation of biomechanical data generally requires the application of numerical modeling approaches.

Numerical modeling provides valuable information on tissue material properties and determining relevant physiological parameters. In this regard, the elastic response has been typically modeled as a hyperelastic material (25) through an appropriate definition of the constitutive formulation, which can be based on intrinsic structural tissue properties (i.e. isotropic (26), transversely isotropic (27)). Likewise, suitable constitutive models have been proposed to characterize viscoelastic (28, 29) and active (30) responses, considering inherent effects on biological tissues, such as mechanical damage (31, 32) and remodeling (33–35). Additionally, blood vessels are subjected to residual stress, which influences their function by homogenizing the stress level resulting from blood pressure (23, 36); however, this factor has been scarcely addressed in umbilical and placental biomechanics studies (37). The level and distribution of this mechanical-type parameter have been suggested as a biomarker for evaluating potential alterations in physiological function (38). Therefore, a key challenge lies in accurately integrating residual stress into numerical simulations to reflect physiological conditions better and improve the identification of early biomechanical markers of vascular dysfunction. This work aims to characterize biomechanical changes in umbilical and chorionic arteries by applying enhanced mathematical modeling, thereby improving the understanding of how fetoplacental circulation may be affected during the progression of FGR.

## Results

### Maternal and newborn parameters and fetoplacental artery morphometry

The characteristics related to the maternal and newborn parameters are exhibited in Table 1. Birth weight (2,602 ± 118.3 g) and height (46.4 ± 0.8 cm) were decreased among FGR group compared with control (3,224 ± 103.7 g; 50.4 ± 0.6 cm, respectively). These maternal and newborn characteristics recapitulate essential features of FGR pregnancies.

**Table 1:** Maternal and neonatal characteristics.

	Control	FGR	*P*-value
Maternal age (years)	28 ± 2.5	32.0 ± 1.4	0.52
Gestational age (weeks)	38.9 ± 0.4	38.2 ± 0.5	0.57
Birth weight (g)	3,224 ± 103.7	2,602 ± 118.3	*<*0.05*
Height (cm)	50.4 ± 0.6	46.4 ± 0.8	*<*0.05*
Percentile range	10–90	2–10	
Gender (*F*/*M*)	1/4	2/3	>0.99
Delivery (*C*/*V*)	2/3	3/2	>0.99

*F* and *M* indicate the total number of female or male neonates, respectively. *C* and *V* indicate the number of cesarean or vaginal deliveries, respectively. Values are expressed as mean ± SEM or frequency. *P < 0.05 determined by Mann–Whitney *U* test or Fisher's exact test (gender and delivery).

Table 2 summarizes the morphometrical analysis of samples. In all parameters measured, umbilical arteries under FGR conditions exhibited differences compared with the control group. FGR samples showed lower wall thickness, along with a decreased relative percentage of inner media area. Conversely, the arteries under FGR conditions showed an increased relative percentage of total outer media area compared with the control. Conversely, FGR chorionic arteries showed no morphometric differences compared with the control.

**Table 2:** Morphological parameters of umbilical and chorionic arteries.

	Control	FGR	*P*-value
Umbilical artery			
Equivalent thickness (mm)	0.25 ± 0.02	0.17 ± 0.02	*<*0.05*
Inner layer (% area)	37.6 ± 1.9	26.7 ± 3.2	*<*0.05*
Outer layer (% area)	62.4 ± 1.9	72.9 ± 3.6	*<*0.05*
Chorionic artery			
Equivalent thickness (mm)	0.18 ± 0.03	0.22 ± 0.01	0.22
Media layer (mm^2^)	0.09 ± 0.10	0.10 ± 0.02	0.88

Values are expressed as mean ± SEM. *Significant difference *P* < 0.05 vs. control. Mann–Whitney *U* test.

### Ring-opening and ring-tensile tests

**Figure 1:**
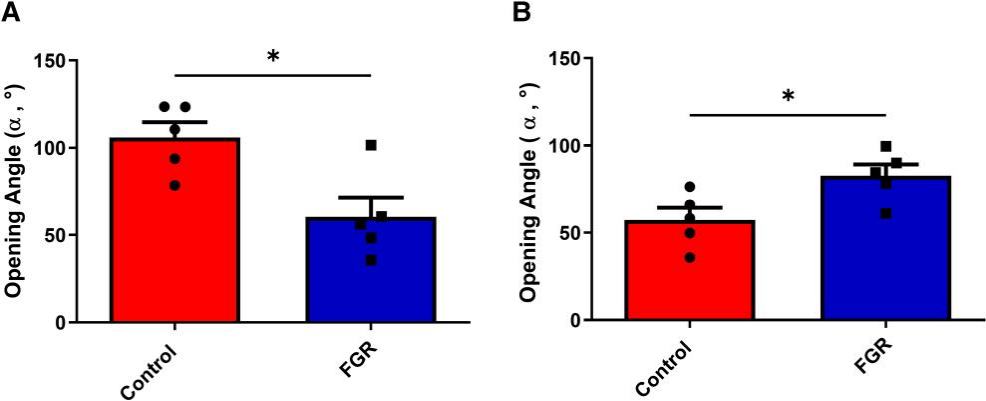
Ring-opening angle test. Opening angle test for (A) umbilical (control [*n* = 5] red bars; FGR [*n* = 5] blue bars) and (B) chorionic arteries (control [*n* = 5] red bars; FGR [*n* = 5] blue bars). Individual values are represented by black dots within both groups. Values are mean ± SEM. * *P* < 0.05 Mann–Whitney *U* test.

**Figure 2:**
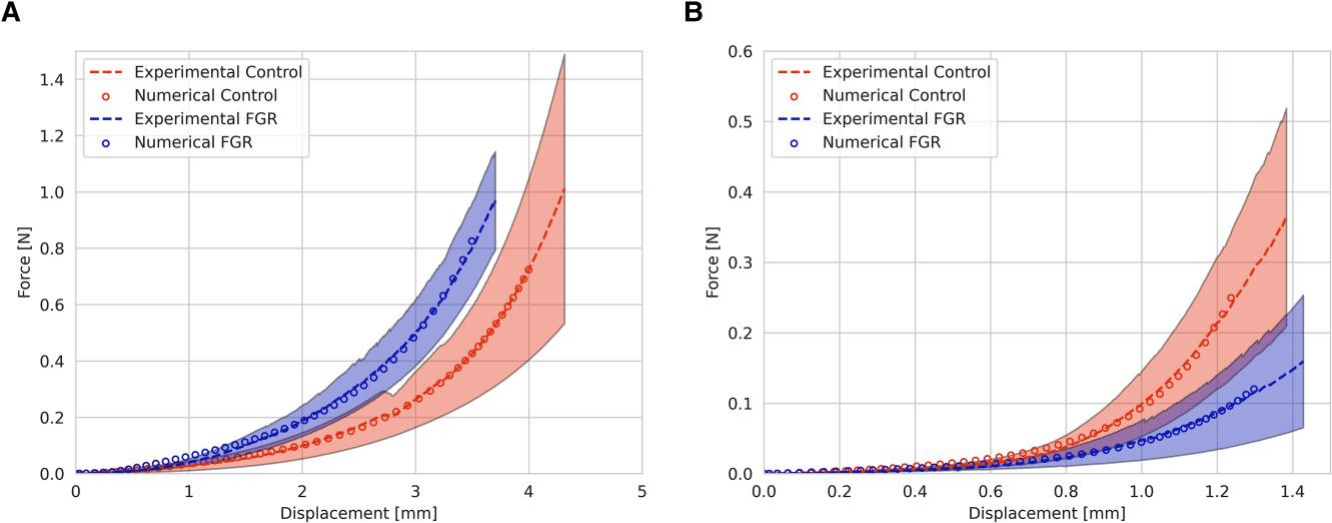
Force–displacement curves in human fetoplacental arteries. Force–displacement experimental (dash line) and numerical calculation (circles) for (A) umbilical and (B) chorionic arteries for control (red; *n* = 5) and FGR (blue; *n* = 5) pregnancies. The values shown represent mean values, and the colored shadow represents the standard error of the mean.

Figure 1 displays the opening angle (*α*) of both arteries studied. Umbilical arteries (Fig. 1A) from FGR pregnancies showed a decreased opening angle (60.5 ± 11.1°) compared with vessels from control pregnancies (106.0 ± 8.8°; *P* < 0.01). In contrast, chorionic plate arteries (Fig. 1B) from FGR pregnancies showed a greater opening angle (82.7 ± 6.4°) compared with control (52.3 ± 6.9°; *P* < 0.05). Figure 2 shows the force–displacement curves obtained from the ring-tensile test for both umbilical (Fig. 2A) and chorionic arteries (Fig. 2B) in the two study groups (i.e. FGR and control). The force–displacement data have no interpretation by themselves, due to the geometrical dependence on both variables. However, this information was used to determine the hyperelastic parameters, as a comparable measure between the experimental groups.

### Hyperelastic parameter fitting

According to the constitutive modeling procedure and making use of the mean values of the biomechanical results exhibited above, the hyperelastic parameters of the Demiray model are exhibited in Table 3. The final hyperelastic parameters obtained after the iterative process consider the two cases mentioned in the explanation of the fitting process: the first, without the presence of the residual stresses when the ring-tensile test was simulated; the second, considering the effect of residual stresses. For both cases, the percentage differences between the Demiray's parameters do not exceed 20%. The final hyperelastic parameters of the Demiray model (with residual stress) are used to generate the force–displacement curves of the ring-tensile test. *R*^2^ indicates a good correlation between experimental and numerical results, where in all cases, these values are >0.99. In Fig. 8B and C, numerical results of the first invariant of the Cauchy stress and the Lagrangian strain along the *Y* direction are presented for both arteries, under FGR conditions, and their corresponding control groups.

**Table 3:** Material parameters of the hyperelastic model.

	Without residual stress		With residual stress	
	Control	FGR	Control	FGR
Umbilical artery				
*a* (kPa)	36.57	69.97	35.67	68.79
*b*	0.53	0.56	0.58	0.59
*R*^2^	0.999	0.997	0.999	0.997
Chorionic artery				
*a* (kPa)	20.70	10.00	17.83	9.91
*b*	2.15	1.08	2.50	1.29
*R*^2^	0.993	0.996	0.993	0.996

### Residual stress

Table 3 exhibits the residual stress values both in the inner and outer zones of the arterial wall, when the closed-ring simulation was applied, following the numerical simulation procedure described in the Materials and methods section. For this purpose, the mean values of the opening angle along with the final hyperelastic parameters of the Demiray model that considers the effect of residual stress (shown in Fig. 1 and Table 3) were used as input for the determination of the residual stress field.

**Figure 3:**
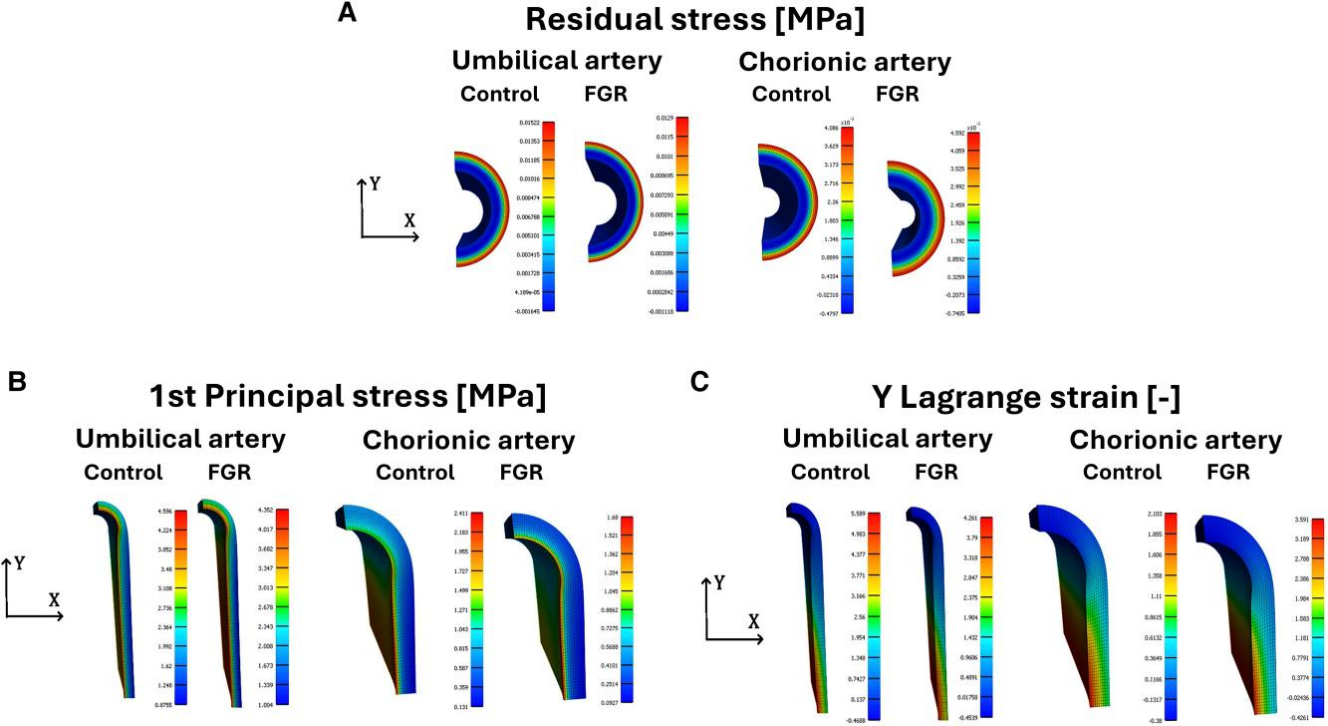
Results obtained via numerical simulation of umbilical and chorionic arteries for both groups studied (control and FGR): A) residual stress, B) first principal Cauchy stress, and C) Lagrange strain along *Y* direction.

Table 4 exhibits the representative results, both in the inner and outer layers of the different ring specimens, whose zones are exhibited in the Materials and methods (Fig. 8). Representative numerical results illustrating the spatial distributions of stresses are provided in Fig. 3A. In umbilical arteries, the control group exhibits higher residual stress in both compression and tension compared with FGR-affected vessels. Conversely, residual stress values in chorionic arteries are lower in the control group than in the FGR group, indicating differential vascular remodeling under FGR conditions.

**Table 4:** Residual stress values in the inner *σ*_in_ and outer *σ*_out_ radius of the arterial ring.

	Control	FGR
Umbilical artery		
*σ*_in_ (kPa)	−16.2	−14.3
*σ*_out_ (kPa)	+15.2	+12.9
Chorionic artery		
*σ*_in_ (kPa)	−4.7	−5.3
*σ*_out_ (kPa)	+4.1	+4.6

The values displayed correspond to the first principal Cauchy stress.

## Discussion

This study presents a comprehensive biomechanical characterization of umbilical and chorionic plate arteries, representing the fetoplacental circulation, with the aim of improving the understanding of how this vascular bed may be affected during the progression of FGR. In this context, differential effects on the mechanical response were observed between the two arterial types. Umbilical arteries exhibited alterations in structural and biomechanical properties, including increased material stiffness and reduced residual stress. In contrast, chorionic plate arteries displayed predominantly biomechanical changes, characterized by reduced material stiffness and increased residual stress. Together, these findings are suggested to reflect distinct remodeling processes within the fetoplacental vasculature in FGR, with structural stiffening predominating in umbilical arteries and biomechanical adaptation occurring in chorionic plate arteries.

The biomechanical characterization of vessels within the fetoplacental circulation remains limited, with most studies focusing on systemic arteries such as the aorta, carotid, and femoral arteries (12, 16, 20, 30). In contrast, investigations of umbilical arteries have reported increased stiffness in ovine models of FGR (21) and in preeclampsia (39). This gap is even more pronounced for chorionic arteries, for which only one biomechanical study has been reported to date (22). Here we characterized the fetoplacental arterial bed involving the umbilical artery to the chorionic plate arteries in control and FGR pregnancies, showing that changes in morphometry, residual stress, and material properties may jointly affect placental pressure and pulsatility. Such an approach allows inference of FGR-related alterations not only in stiffness but also in mechanical mismatch between vessel segments (40), with potential consequences for downstream perfusion, and provides parameters suitable for integration into hemodynamic simulations and Doppler-based assessments (41). Accordingly, we adopted a finite element framework consistent with established approaches for simulating arterial ring-tensile tests (38, 42), closely aligned with the methodology of Mahutga et al. (43). In that framework, force-displacement data are used to identify hyperelastic material parameters, offering advantages over stress–strain formulations due to their direct experimental basis and reduced sensitivity to geometric uncertainty. While Mahutga et al. (43) neglected residual stress, the present study extends this approach by incorporating residual stress derived from ring-opening tests. This addition allows a more physiologically representative description of the arterial wall, which is known to retain intrinsic stress in the unloaded state (44) and enables a more comprehensive biomechanical characterization of fetoplacental arteries under pathological conditions such as FGR.

Demiray material parameters for the umbilical artery were higher in the FGR group when residual stress was included, suggesting increased circumferential stiffness. This finding is consistent with previous reports in FGR sheep (21) and in human umbilical arteries under preeclampsia (39), which have linked increased stiffness to alterations in elastic fibers, collagen content, and related microstructural components. However, studies in smaller animal models, such as FGR guinea pigs, have reported no changes in umbilical artery material properties (11), suggesting species-related differences. In contrast, chorionic arteries exhibited reduced stiffness in FGR, which differs from the increased stiffness reported by Saw et al. (22). Notably, that study also described a reduced collagen-to-elastin ratio under severe FGR, consistent with an increased elastin contribution and supportive of our findings regarding elastic fiber–dominated mechanical behavior.

Residual stress is a key biomechanical parameter that has been identified as a potential biomarker for assessing vascular remodeling in the cardiovascular system (44). In the case of the umbilical artery, residual stress levels were markedly lower in the FGR group compared with controls. This behavior has already been observed in the umbilical arteries (20), where the differences in the residual stress levels were attributed to a reduced quantity and disorganization of elastic fibers. Conversely, in the chorionic arteries, the residual stress field was higher for the FGR group. According to our search, only one previous study has applied the ring-opening test in chorionic arteries (22), which does not quantify the residual stress levels. This increase is consistent with the higher elastin content observed in the arterial wall. In umbilical arteries, Dodson et al. (21) reported an increase in collagen fibers within the outer layer under FGR conditions. Similarly, we found a higher relative percentage of the outer layer in the FGR group compared with the control group. This increase potentially suggests a greater collagen content (45). In addition, Dodson et al. (21) also reported a greater glycosaminoglycan (GAG) content in the media layer of umbilical arteries. GAGs contribute to increased stiffness by reinforcing the collagen network (46), further supporting our evidence of elevated vascular stiffness associated with FGR. Collectively, the evidence indicates that FGR induces both growth and arterial remodeling processes.

In contrast, no differences were observed in chorionic arteries’ wall thickness. These results are consistent with findings by Saw et al. (22), who reported no significant changes in the vascular radius of chorionic arteries under FGR conditions. Consequently, the morphological and biomechanical observations from our study, combined with previous evidence of a relative increase in elastic fibers (22), suggest that vascular remodeling in chorionic arteries occurs without growth-related changes. The incorporation of residual stress may improve the modeling (23). Although there are studies that include its effect (11), there are also several works that do not include it (43, 47), which may explain some differences. Altogether, the differing mechanical responses observed in the umbilical and chorionic arteries imply that vascular adaptation in FGR may occur through separate pathways in each vessel type.

We acknowledge several limitations in our study. First, we did not quantify collagen content, which limits our ability to link mechanical changes to specific matrix components. Since extracellular matrix remodeling is central to vascular adaptation, future analyses incorporating histochemical or biochemical assays will be critical to clarify these associations. Moreover, deeper molecular characterization of signaling pathways involved in vessel stiffening may reveal novel therapeutic avenues for FGR and related vascular pathologies in pregnancy. Expanding the sample size in future work will also improve statistical power and enable stratified analyses by gestational age, severity of FGR, or maternal comorbidities, refining our interpretation of the biomechanical findings.

## Conclusions

Altogether, this study highlights differential vascular responses to FGR, suggesting that structural and mechanical adaptations occur independently across fetoplacental vessels despite their anatomical connection. Future work will aim to assess the effect of changes in pregnancy through another characteristic of the mechanical response, such as viscoelasticity (48), contractile response (11), among others. In addition, ultrastructural and molecular assessments, such as histological and immunohistochemical analyses, may support the biomechanical findings and corroborate observations previously reported in the literature.

## Materials and methods

### Ethics and participants

All procedures of this study were performed in accordance with the Declaration of Helsinki. All participants who voluntarily donated placental samples signed informed consent. The study was approved by the ethics committee of the Faculty of Medicine at the Pontificia Universidad Católica de Chile (protocol #170705023). All biological samples and clinical data were coded and anonymized following the acquisition of written informed consent. Neonate weight under the 10% percentile with Doppler alterations (pulsatile index >90%) (8) was considered as a selection criterion for the FGR group in this study. The Ponderal index was computed using the method described by Fay et al. (49). The percentile range was determined using the Alarcon–Pittaluga intrauterine growth curves table as outlined by Milad et al. (50).

### Placental collection and tissue processing

Placenta samples were collected from pregnancies without unusual findings (*n* = 5) and pregnancies with a clinical diagnosis of FGR (*n* = 5) delivered at the Maternity of Clinical Hospital of the Pontificia Universidad Catolica de Chile. The umbilical and chorionic arteries were isolated from the placental tissue, ensuring a distance of ∼2 cm from the umbilical cord insertion. Because both the ring-tensile test and the ring-opening test are destructive procedures, they were performed on adjacent arterial segments extracted from the same artery. To minimize potential inaccuracies arising from spatial heterogeneity of arterial mechanical properties, the ring-opening test and the ring-tensile test were performed sequentially. This experimental strategy aimed to reduce intersample variability while preserving the structural integrity required for each test and has been adopted in previous studies of arterial residual stress estimation (38). The vessels used for the ring-opening and ring-tensile tests were placed in calcium-free Krebs solution containing the following concentrations (in mmol/L): 118.5 NaCl, 25 NaHCO_3_, 4.7 KCl, 1.2 KH_2_PO_4_, 1.2 MgSO_2_, and 5.5 D-glucose. Segments of vessels were randomly designated for histological studies and were immersed in a solution of 4% paraformaldehyde (4%) solution overnight. For the biomechanical testing, the samples were processed without storage, and all experiments were conducted within 3 to 4 h following sample collection.

### Ring-opening test

**Figure 4:**
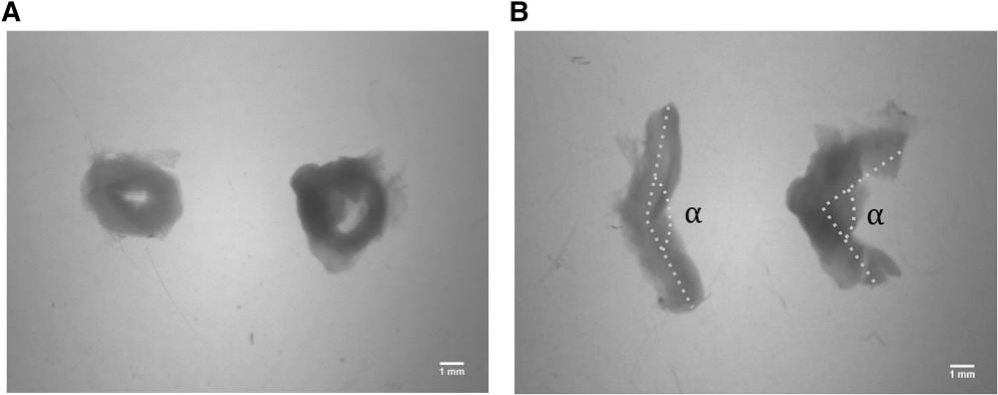
Representative images from the opening angle assay conducted on control umbilical arteries. A) The initial configuration at time = 0 min. B) The configuration after 20 min, when opening angle measurements were obtained. Scale bar = 1 mm.

The ring-opening test allows measuring the opening angle (*α*) formed by the ends of the vessel after a radial cut (Fig. 4). For this purpose, isolated vessels were cut into 2 mm cross-sectional sections and kept for 10 min in Ca^2+^-free Krebs solution at a temperature of 37 ± 1 °C for equilibration (Fig. 4A). Then a radial cut was performed and kept at the same temperature in the same solution for 20 mins. Finally, arterial rings were photographed using a Leica EDHD24 magnifying glass (Leica), and the aperture angles were quantified using the Image J Software (NIH). The angle's vertex was defined as the midpoint between the open ends of the artery, with each end representing one arm of the angle. The angle was measured in degrees (Fig. 4B). These measurements were performed five times in the same sample, and then the values were averaged. This protocol strictly followed the methods previously established (51) and widely used and optimized by our group (16, 38, 52). Measurements were conducted in 5 replicates to obtain an average for each sample considered.

### Ring-tensile test

**Figure 5:**
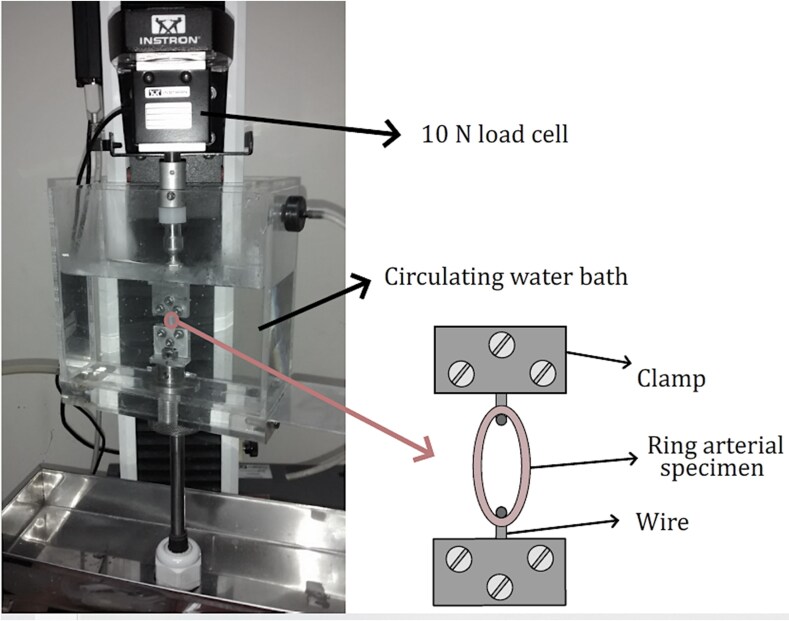
Experimental setup of the ring-tensile test.

The ring-tensile test has been extensively employed to characterize tubular-shaped tissues, particularly when the standard planar tensile test is not feasible, mainly due to size-related restrictions (43). A ring arterial specimen was mounted in the test machine Instron 3342 (equipped with a 10-N load cell), through two tungsten wires that were placed on the inner side of the ring sample, and an initial preload of 0.5 N was applied before the test began. During the test, the tubular sample was subjected to radial elongation, and the instantaneous force–displacement data were registered. Before the test began, the initial vessel dimensions, including thickness (*t*), length (*L*), and internal diameter (*ϕ*_int_), were measured by a glass magnifier, and subsequently measured by images using the software ImageJ (53). Throughout the test, the samples were kept in Ca^2+^-free Krebs solution at a temperature of 37 ± 1 °C, through a circulating water bath. Figure 5 exhibits the experimental setup used in this test.

### Morphology

**Figure 6:**
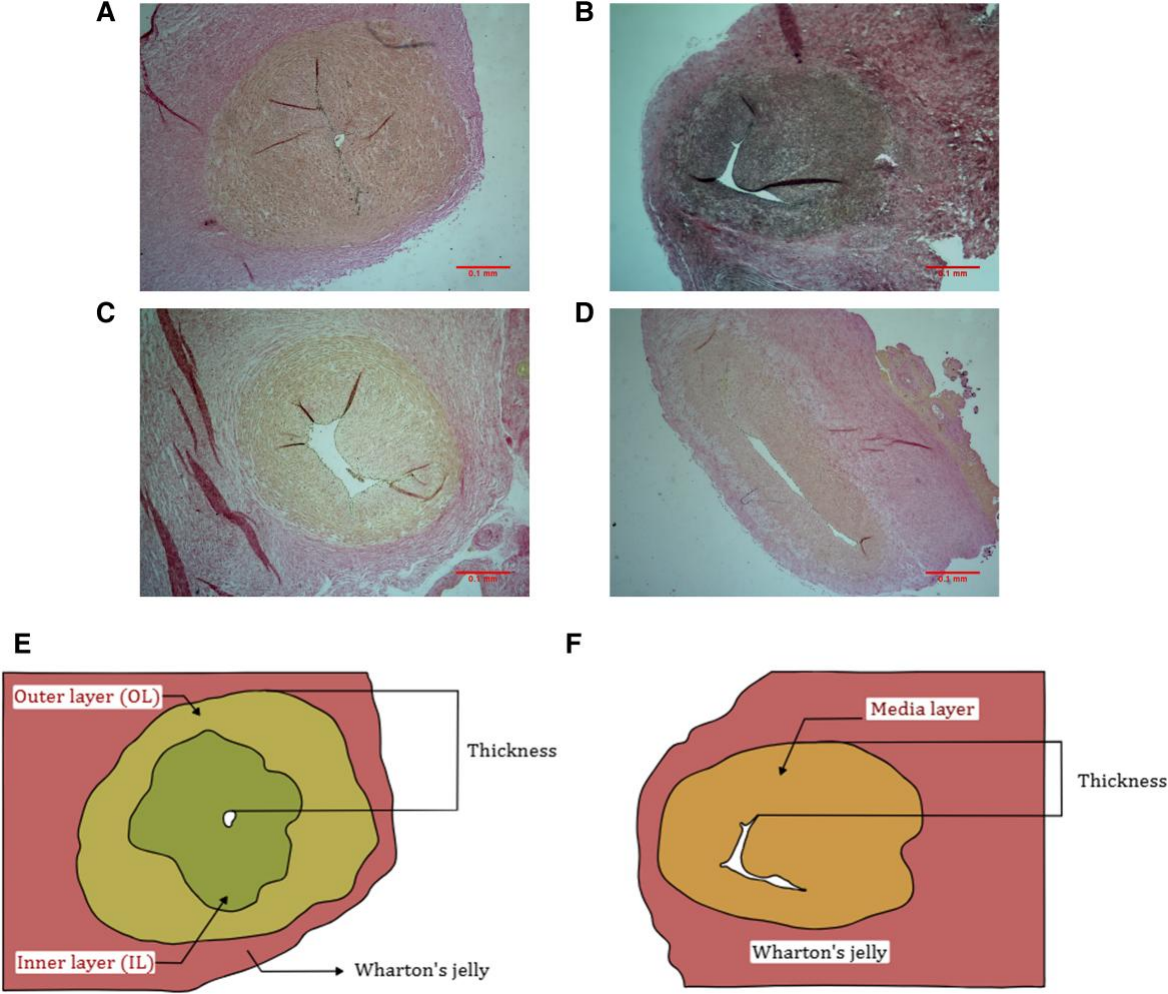
Representative Van Gieson–stained sections of fetal (umbilical) and placental (chorionic) arteries from healthy and FGR pregnancies: A) healthy umbilical artery, B) healthy chorionic artery, C) FGR umbilical artery, and D) FGR chorionic artery. Scale bar: 0.1 mm. Representative scheme showing the procedure applied to measure the morphological parameters for E) umbilical and F) chorionic arteries.

Sections of umbilical artery and chorionic artery with a thickness of 5 µm were mounted on a slide and stained using hematoxylin and van Gieson staining (20). Representative histological images are displayed in Fig. 6A–D. From the images obtained after this procedure, all measurements were conducted with the software ImageJ, and the scheme of the procedure carried out is displayed in Fig. 6E and F. For both arteries, total wall thickness is defined as the distance from the lumen to the outer limit of the vessel (54). To avoid bias due to sample's irregular shape, an average of five measurements was considered in the analysis. Conversely, the quantification of the media layer was performed between the zone media layer area encompassed between the lumen and the adventitia layer was calculated (20). Whereas this quantification is straightly performed for the chorionic arteries, in umbilical arteries the intima-media layer is subdivided into two types of muscle fibers: inner layer and outer layer (55). The arterial wall compartments were quantified histologically using area-based morphometry. For each vessel cross-section, the external boundary of the tunica media was delineated to determine the total area enclosed by the media. The luminal area was traced and subtracted from this value to calculate the total medial area (media area = area within the outer media border minus lumen area). The region of the media with predominantly circumferential cellular orientation was manually identified and measured as the outer media. The remaining medial area (total medial area minus outer media area) was defined as the inner media, corresponding to cells with a more disorganized orientation located closer to the lumen (56). Furthermore, to investigate the possible mechanical impacts of the muscle layer based on its phenotype (either circumferential or synthetic), the percentage of each layer (outer layer = spindle shape; inner layer = irregular shape) was determined by analyzing the alignment of the cells in the artery (55) and therefore, calculating the area for each layer.

### Constitutive modeling

Hyperelastic model: The purely elastic response in soft tissues is often modeled through hyperelastic models (25). Particularly, its definition is stated by an appropriate strain energy density function (*W*), which allows the description of the material behavior under any deformation condition, determined by the deformation gradient tensor **F**. In this work, umbilical and chorionic arteries were modeled by using an exponential-like constitutive model, whose strain energy density *W* as defined by the isotropic Demiray model, whose definition is stated by


(1) $$W=\;\frac{a}{b}\exp\frac{b}{2}\;(I_{1}-3),$$

where the material parameter *a* (kPa) governs the initial stiffness of the tissue and primarily controls the stress response at low strain levels, whereas the dimensionless parameter *b* controls the material's nonlinear stiffening at larger deformations. In addition, *I*_1_ = tr **C** is the first invariant of the right Cauchy–Green tensor (**C** = **F**^T^**F**), whereas the Cauchy stress tensor is obtained using continuum mechanics theory $\sigma \;=\frac{2}{J}\;F\cdot \;\frac{\partial W}{\partial C}\cdot F^{T}$, where **C** is the right Cauchy–Green tensor **C** = **F**^T^  **F**, J = det **F** is the determinant of the tensor **F**. This constitutive model was used in the finite element (FE) context, using the software FEBio (57) for this purpose.

The Demiray model was selected as a phenomenological constitutive formulation suitable for describing the purely elastic response of soft biological tissues under finite deformations. Comprehensive reviews of hyperelastic energy density functions recognize this model as an appropriate isotropic representation when experimental information is limited to a single loading mode (58). Experimental data correspond to mechanical responses measured along a single loading direction, which would render more complex anisotropic formulations underdetermined. For this reason, the isotropic Demiray model was adopted as a modeling assumption consistent with the scope and nature of the available data, acknowledging this as a limitation of the study. The applicability of the Demiray model to biological tissues has been supported by previous experimental and computational works, including studies in cardiac electromechanics and soft tissue elastography (59, 60).

### Fitting of material parameters

**Figure 7:**
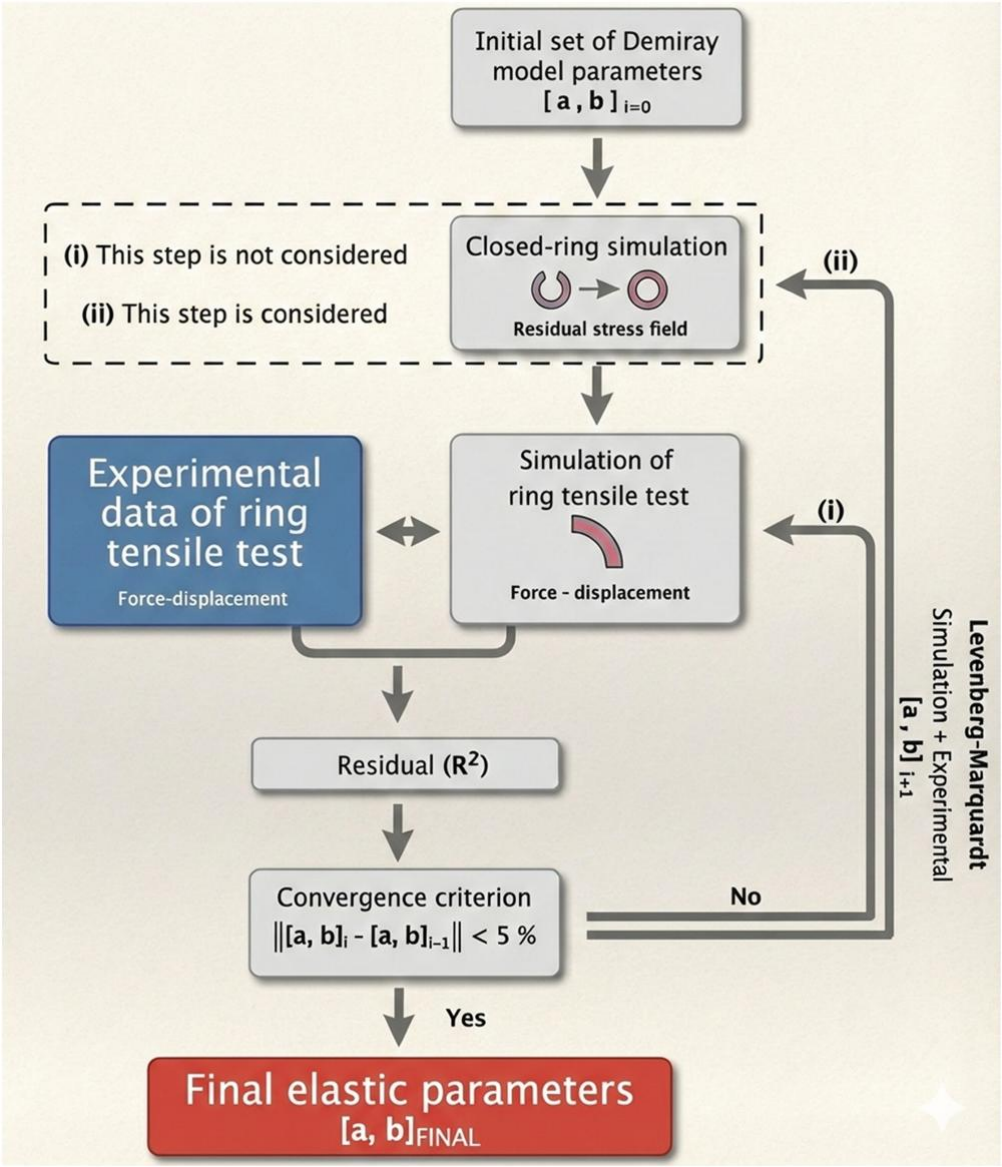
Graphical workflow for algorithms employed to the material parameters fitting.

Hyperelastic model: Material parameters of the Demiray model (parameters *a* and *b*) were identified through an optimization procedure combining experimental biomechanical data with corresponding numerical simulations. The parameter-fitting workflow is schematized in Fig. 7. Two established approaches were implemented: (i) neglecting residual stresses in the ring specimen during tensile testing (43) and (ii) accounting for residual stresses (11). Comparing these approaches allows assessment of the influence of residual stress on the estimated material parameters.

Parameter identification was performed using a group-level approach. For each artery type and experimental group, individual force–displacement curves were first interpolated onto a common displacement domain and then averaged to obtain a representative group-level response. An averaged geometry, obtained from the corresponding geometric measurements within each group, was used in the numerical model. The constitutive model was then fitted once per group using the averaged force–displacement curve and averaged geometry, allowing comparison between groups through material parameters interpreted as representing the average tissue response. The fitting technique employed was a nonlinear least-squares method implemented using a Levenberg–Marquardt optimization algorithm, in which the Jacobian matrix is approximated using a finite-difference approach, as available in the Python’s LMFit package. Material parameters of the Demiray model were identified by minimizing the residuals between experimental and numerical force–displacement responses, where the objective function is defined as *ϕ*(**θ**) = 0.5 ∑*_i_*  _=_  _1…*N*_[ = *F*^sim^(*d_i_*, **θ**) − *F*^exp^(*d_i_*)]^2^, with *F*^exp^(*d_i_*) and *F*^sim^(*d_i_*, **θ**) denoting the experimental and simulated forces at the displacement level *d_i_*, respectively, *N* the number of experimental data points, and **θ**={*a,b*} the vector of Demiray material parameters. The goodness of fit for each material group model fitting was evaluated using the coefficient of determination (*R*^2^). The fitting of material parameters was performed within each experimental group.

### Numerical simulation

**Figure 8:**
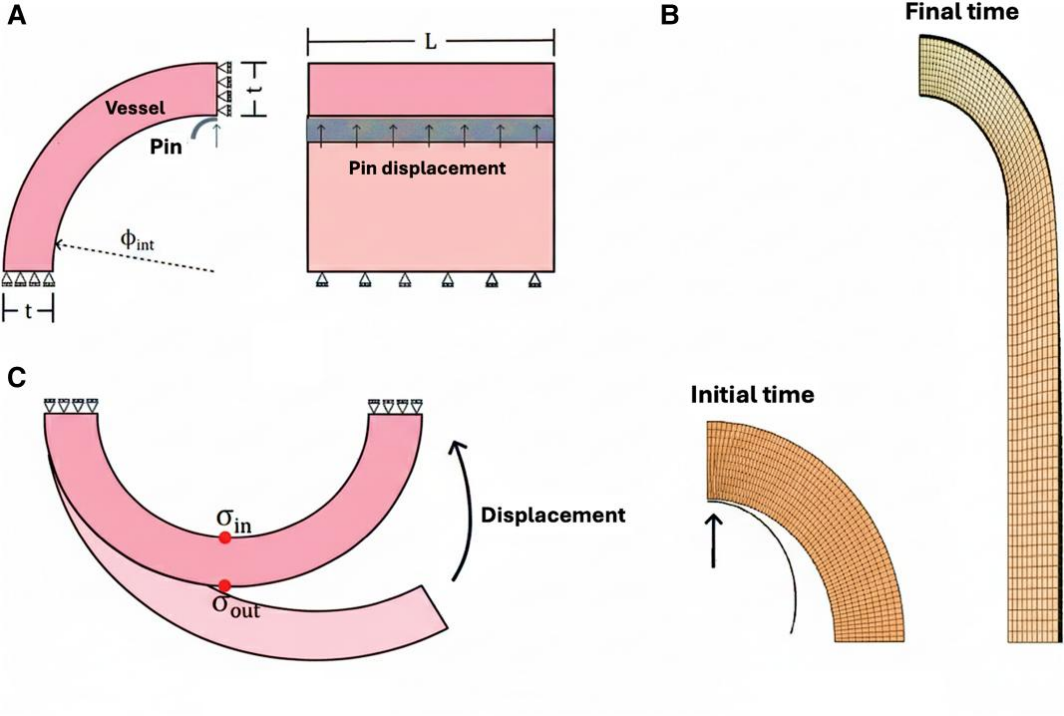
Summary of ring-tensile test simulation conditions. A) Geometry and boundary conditions of the ring-tensile test simulation. B) Deformed configuration of the ring-tensile test simulation. C) Numerical scheme of the residual strain reconstruction, identifying the regions from which the residual stresses (*σ*_in_ and *σ*_out_) were obtained.

All simulations were conducted using FEBio software (Finite Elements for Biomechanics), version 4.7 (57). The computational modeling of the ring-tensile test (Fig. 8A) considers a quarter of a ring geometry (defined by internal diameter *ϕ*_int_, thickness *t*, and length *L*) subjected to symmetrical boundary conditions. The average dimensions of the initial arterial ring geometries, used in the numerical simulations, were as follows: umbilical artery—control: *ϕ*_int_ = 0.94 mm, *t* = 0.25 mm, *L* = 2.46 mm; umbilical artery—FGR: *ϕ*_int_ = 0.97 mm, *t* = 0.19 mm, *L* = 2.88 mm; chorionic artery—control: *ϕ*_int_ = 0.58 mm, *t* = 0.16 mm, *L* = 2.80 mm; and chorionic artery—FGR: *ϕ*_int_ = 0.43 mm, *t* = 0.18 mm, *L* = 2.27 mm. Although the umbilical and chorionic arteries exhibit irregular thickness and non-uniform cross-sectional geometry, the arterial wall was modeled as an axisymmetric ring. This assumption was adopted as a first-order approximation toward a patient-specific geometrical representation, allowing capture of the dominant mechanical response while maintaining numerical robustness for constitutive parameter identification. Axisymmetric formulations are commonly employed in arterial wall modeling, particularly in group-level analyses of vascular mechanics (61). Nevertheless, this simplification neglects local geometric heterogeneities, which may influence regional stress and strain distributions, and this limitation should be considered when interpreting the numerical estimates.

The finite element mesh consisted of ∼24,000 eight-node hexahedral elements with full Gauss integration (2 × 2 × 2), refined in regions of high deformation. A typical deformed configuration is shown in Fig. 8B. A three-field formulation was employed to account for the quasi-incompressible behavior of the uncoupled material model (62). A rigid wire, modeled with 2,700 quadrilateral elements, was displaced along the *y*-axis. Contact between the wire and the vessel's inner surface was defined using FEBio's sliding-elastic formulation (63), with enforcement via an Augmented Lagrangian scheme, an automatic penalty calculation, a nonsymmetric stiffness formulation, and a gap tolerance of 1e^−3^ mm. Frictional effects were neglected. Residual stress was incorporated using the prestrain algorithm implemented in FEBio, which introduces a prescribed prestrain gradient tensor (**F**_p_) to generate a corresponding prestress state. To ensure compatibility with the reference geometry and boundary conditions, the prestrain tensor is iteratively updated following $F_{p}^{k+1}=(F^{k}{)}^{-1}F_{p}^{k}$ until the distortion falls threshold of 0.01. The initial deformation gradient field used for prestraining was obtained through a simulated ring-closure process (Fig. 8C). Beginning from a stress-free open-ring configuration, displacements under rigid-body constraints were applied to bring the vessel ends together. Upon reaching mechanical equilibrium, the deformation gradient tensor field was extracted. To transfer this field onto the ring mesh used in the tensile test, interpolation was performed using the pyvista Python package (64). The prestrain was applied element-wise and introduced progressively over 20 loading steps, increasing linearly from zero to the final deformation state. Following this, the rigid shell was displaced along the *y*-axis. A numerical solution to the problem was carried out using a Broyden quasi-Newton scheme, with up to 15 updates before reassembling the stiffness matrix. An automatic time-stepper controlled the simulation progress, allowing between 200 and 500 total steps. The force–displacement response was extracted using a custom Python binary reader for FEBio's.xplt output format.

### Statistical analysis

All data of the samples were expressed as mean ± SEM, calculated as the ratio of the SD to the square root of the number of specimens (*n*). The representation of the mean population differences is quantified through the statistical confidence interval (represented by the *P*-value). To this aim, a well-defined procedure was applied to determine the statistical test considered (17). In all cases, a nonparametric Mann–Whitney *U* test was used for the comparison between two groups. Fisher's exact test was used when appropriate (discrete variables). Differences were considered significant when *P*-value was ≤0.05. The software GraphPad Prism 9.01 (GraphPad Software Inc., San Diego, CA, United States) was used for this purpose.

## Supplementary Material

pgag158_Supplementary_Data

## Data Availability

Data used in the study are included in the manuscript and in the Supplementary material.
